# Comparison of milk production and milk composition for an exotic and a local synthetic rabbit lines

**DOI:** 10.14202/vetworld.2017.526-529

**Published:** 2017-05-15

**Authors:** Karim El-Sabrout, Sarah Aggag, Alaa El-Raffa

**Affiliations:** 1 Department of Poultry Production, Faculty of Agriculture (El-Shatby), P.O. Box 21545, University of Alexandria, Alexandria, Egypt; 2 Department of Genetics, Faculty of Agriculture (El-Shatby), P.O. Box 21545, University of Alexandria, Alexandria, Egypt

**Keywords:** Alexandria line, milk yield, proteins, rabbit performance, V-line

## Abstract

**Aim:**

Basic objective of this research was to compare two rabbit lines: V-line (exotic line), Alexandria (local synthetic line) for yield and composition of milk product and their effect on productive performance of rabbits.

**Materials and Methods:**

The experiment was conducted on 80 does and their kits. Milk yield (MY) of each doe and milk composition (MC) were recorded at 3^rd^ week of lactation.

**Results:**

The results of MY showed insignificant difference between V-line and Alexandria line. While the results of MC indicated significant differences in milk protein and fat between the two lines. The protein electrophoresis was used for assaying variation in milk proteins between the two lines. The banding protein patterns showed seven protein bands for Alexandria line and six bands for V line. The results demonstrated one specific protein marker at 48 KDa (κ-casein) in Alexandria doe’s milk. Moreover, the results of individual body weight at weaning age indicated that Alexandria rabbits had significantly higher body weight compared with V-line rabbits (845.33 g, 664.05 g, respectively). Alexandria line had significantly lower mortality rate compared with V-line (1.5%, 2.7%, respectively).

**Conclusion:**

The differences which obtained in Alexandria line milk may play an important role in the productive performance of rabbits.

## Introduction

Milk is usually the sole source of nourishment of young mammals. Growth and survival of rabbit kits depend exclusively on the doe’s milk production as a first food for the newly born offspring [1]. Milk traits performance such as milk production, composition, and physical properties are influenced by genetic [2].

V-line is a synthetic maternal line originated at the Department of Animal Science, Politecnica University, Valencia, Spain [3]. Alexandria line is a synthetic paternal line which comes from the crossing of V-line (maternal line) with Black Baladi (paternal line) rabbits [4]. However, the nutritional value of milk is closely related with its composition, and it is associated with growth and development of young that certainly are used as selection criteria. Therefore, studying the milk protein polymorphism is very importance in animal breeding [5]. Protein polymorphism provides beneficial information such as the relationships between rabbit genotypes and their biochemical expressions [6].

The objective of this study was to determine the differences between V-line and Alexandria line for yield and composition of milk, which can influence the productive performance of rabbits.

## Materials and Methods

### Ethical approval

The approval from the Institutional Animal Ethics Committee to carry out this study was not required as no invasive technique was used.

### Study design

V-line and Alexandria line rabbits have been used from the stocks available at rabbitry of the Poultry Research Center of Alexandria University, during season 2015/2016. The experiment was conducted on 80 does (40 does from each line) and their kits (478 V-line and 469 Alexandria). Rabbits were housed in cages and fed commercial pelleted diet (18% protein and 2600 Kcal/kg). Breeding rabbits (does and bucks) were kept individually, and they were offered *ad libitum* diet until 4 months of age and then they received a restricted quantity (about 140-150 g/day) until the first mating. The first mating has been in average of 5 months of age. The breeding rabbits were divided into families; each family was made by four does and one buck (sex ratio) that was chosen to avoid breeding of close relatives relationships. At the mating time, each doe was transferred to the buck’s cage, and they left with bucks about 10-15 min after success of the first copulation and then returned to their cage. In general, does were remating 10 days after parturition (semi-intensive rhythm). Milk yield (MY) (40 does from each line) was recorded as an average during the 3^rd^ week of lactation (peak of milk production) for the weight of both doe and kits before and after suckling. Furthermore, 40 milk samples from each line were collected manually for milk composition (MC) records by gently massaging the mammary gland after 3 min of injection does with 0.5 ml of oxytocine hormone to stimulate the milk ejection. Milk samples were firstly defatted (the cream is removed from whole milk) by centrifugation at 6000 rpm at 4°C for 20 min, resulting in the cream layer (at the top) and the skim milk (milk plasma). The skim milk samples were transferred (cooling) immediately to the laboratory for the chemical analyses. Rabbit MC (protein, fat, and lactose) was determinate (%) by infrared absorption instrument (MilkoScan Foss Electric). Ash was determined (%) according to the procedure outlined in AOAC [7].

Milk proteins were further separated by sodium dodecyl sulfate-polyacrylamide gel electrophoresis (SDS-PAGE). 15% polyacrylamide gel was prepared by mixing 3.33 ml of 30% acrylamide (acrylamide + bis acrylamide 30: 0.8), 5.0 ml of 1.5 M Tris-HCL (pH 8.8), 50 µl of 10% ammonium per sulfate, 4.02 ml of distilled water, and 10 µl tetramethylethylenediamine. 10 µg of protein samples diluted in migration buffer (1.5 M Tris-HCl pH 6.8, 17.4% glycerol, 8% SDS, 0.08% bromphenol blue and 3M beta-mercaptoethanol) were migrated for 1 h and 45 min using Mini-Protein II vertical cell (Bio-Rad, USA) at 75 V in the beginning followed by 125 V to the end of electrophoresis (2 h). After migration, gels were stained by immersion in staining dye (Coomassie Blue 250 R, USA) for 15 min and distained with 7% acetic acid solution for 3 min and then washed with distilled water. The resolved protein bands were subjected to analyze with the TotalLab software version 13 for Windows (USA). Moreover, data were collected on individual body weight (g) at 28 days (weaning) (BW1), individual body weight (g) at 63 days of age (market age) (BW2), litter size (LS) at birth and litter mortality rate (%) from birth to market (M).

### Statistical analysis

Data recorded are expressed as mean±standard error of the mean. Statistics differences between the two lines were determined by ANOVA using the general linear model from the SPSS statistical program [8] followed by Duncan’s multiple range test. The following statistical model was used:

Y_ijfkhmnl_=µ+G_i_+P_j_+S_f_+BW1_k_×LS+BW2_h_+MY_m_× LS+MC_n_+e_ijfkhmnl_

Where, Y is the dependent variable; µ is the overall mean of observations; G is the fixed genotype effect; P is the fixed parity effect; S is the fixed season effect; BW1 - Individual body weight at weaning, BW2 - Individual body weight at market, MY - Milk yield and MC - Milk composition are the covariates by LS - Litter size and e is the residual error.

## Results

**Figure-1:**
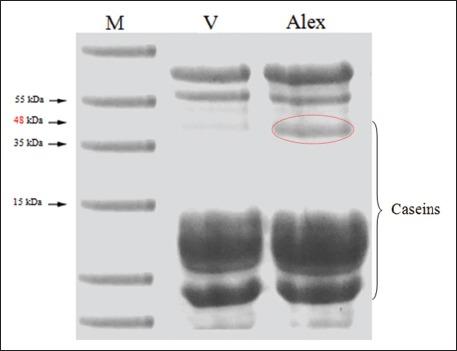
Sodium dodecyl sulfate-polyacrylamide gel electrophoresis separates milk proteins of V-line (V) and Alexandria (Alex) line. M=Marker.

From the results of MY recorded during the 3^rd^ week of lactation (Table-1), we did not find a difference between V-line and Alexandria line. The LS at birth was close between the two lines does, which means that it had no effect on MY. Analysis of the two lines MC included protein, fat, lactose, and ash was determined (Table-2). Significant differences (p≤0.05) in content of milk protein and fat were found while no significant differences in lactose and ash were found. Skimmed milk samples were further analyzed for protein polymorphism. Scan of SDS-PAGE patterns of proteins for V-line and Alexandria line shown in Figure-1. The banding protein patterns showed seven protein bands which are diagnostic to Alexandria line, while six bands are detected for V-line. The results revealed one specific band at 48 KDa (κ-casein) which could be used as specific protein marker to characterize Alexandria rabbits. There were several types of proteins in milk. The primary group of milk proteins was the caseins (about 75% including αs1-casein, αs2-casein, β-casein, and κ-casein) which have an appropriate amino acid composition for growth and development of the young. Other milk proteins were grouped together under the name of whey proteins which included variants proteins such as growth factors, enzymes, and immunoproteins. The major whey proteins in rabbit milk were α-lactalbumin and β-lactoglobulin. Protein electrophoresis technique used in this study provides an accurate technique for determining variation in milk proteins. The milk protein profile of the two lines showed the presence of the major caseins variants: αs1-casein, αs2-casein, β-casein, κ-casein (only for Alexandria) and two whey proteins: α-lactalbumin and β-lactoglobulin (Figure-1). The different isoforms of caseins were identified according to their molecular weight values. Analyses of the different types of milk protein revealed that the highest expression was observed for β-casein and followed by αs1-casein, αs2-casein. In addition, there was higher expression observed for β-lactoglobulin than α-lactalbumin.

**Table-1:** Means and standard errors of LS and MY for V-line and Alexandria line rabbits during the 3^rd^ week of lactation.

Lines	n	LS	MY (ml/day)
V-line	40	8.9±0.10	243±0.01^NS^
Alexandria	40	8.4±0.11	239±0.01

NS=Not significant, MY=Milk yield, LS=Litter size

**Table-2:** Means and standard errors of MC for V-line and Alexandria line rabbits during the 3^rd^ week of lactation.

Lines	n	Protein (%)	Fat (%)	Lactose (%)	Ash (%)
V-line	40	8.41±0.08^b^	11.03±0.19^b^	2.58±0.07^NS^	1.83±0.02
Alexandria	40	10.50±0.12^a^	13.78±0.22^a^	2.51±0.05	1.88±0.04^NS^

a,b Means in the same column with different superscripts are significantly different (p≤0.05). NS=Not significant, MC=Milk composition

Means and standard errors of individual body weight at weaning age (28 days), individual body weight at market age (63 days) and mortality rate of V-line and Alexandria line rabbits shown in Table-3. The results indicated that weaning body weight in Alexandria line was about a 21% higher (p≤0.01) than V-line rabbits. After then, we did not find a difference in body weight (market age). Furthermore, Alexandria line was lower (p≤0.05) in the mortality rate compared with V-line (1.5%, 2.7%, respectively).

**Table-3:** Means and standard errors of individual body weight at weaning (28 days), individual body weight at market (63 days) and mortality rate (%) of V-line and Alexandria rabbits.

Lines	BW1 (g)	BW2 (g)	M (%)
V-line	664.05±10.62^b^	1455.12±17.01	2.7^a^
Alexandria	845.33±11.21^a^	1520.31±18.20^NS^	1.5^b^

a,b Means in the same column with different superscripts are significantly different (p≤0.05, 0.01). NS=Not significant, BW1=Individual body weight at weaning (28 days), BW2=Individual body weight at market (63 days), M=Mortality rate (%) from birth to marketing

## Discussion

The essential elements in litter growth and mortality rates during the suckling period are extremely dependent on doe milk. Rabbit studies relating the milk production and composition to productive traits are minimal. The previous studies confirmed this positive phenotypic relationship between MY and LS in rabbits [9,10]. In this study, we did not find a difference in LS between the two lines. Rabbit milk production may be affected by line of doe [10].

In general, rabbit milk production shows gradual increase until the 20^th^ day of lactation. After then, it decreases by the next days [11]. Results of MY recorded during the 3^rd^ week of lactation between the two lines were close. This may be due to genetic structure of Alexandria that included maternal line (V-line). These results also were close to the findings of Volek *et al*. [12] working on Hyplus rabbits (exotic line) and Abou Khadiga *et al*. [13] working on APRI rabbits (local line).

Composition of rabbit milk affected by many factors such as genotype [14]. To investigate the difference in MC between two lines rabbit does, basic analysis of milk was performed at the same conditions. In our study, significant differences in content of milk protein and fat were found between V-line and Alexandria line, while no significant differences in lactose and ash content of milk were found. On the other hand, Maertens *et al*. [15] found no significant differences in MC traits between commercial hybrids.

The genetic polymorphism of rabbit milk proteins was also investigated in this study as being of great importance in breeding. Variation in protein profile reflects changes in the genes that code for them. The protein banding pattern of each genotype reveals a biochemical genetic fingerprint and each band in the pattern reflects a separate transcriptional level. Some previous studies suggest that milk protein polymorphism has a strong influence on milk qualitative and quantitative traits [16,17]. In addition, the electrophoretic method is an accurate applied to detect milk protein polymorphism between lines.

κ-casein (phospho-protein) determined in Alexandria line milk is an expression of gene which involved in a number of important physiological processes. It is responsible for increased efficiency of digestion [18]. The results indicated that body weight at weaning age in Alexandria line was higher than V-line rabbits. The Alexandria line was selected for daily weight gain [4]. However, the closely post-weaning body weight observed between V-line and Alexandria line rabbits may be due to separate young rabbits from doe milk and intake the same diet till market.

## Conclusion

From the results of this study, we can conclude that there were differences between the V-line and Alexandria line for MC. The specific protein marker (κ-casein) which obtained in Alexandria line milk may play an important role in the productive performance of rabbits. Further researches with large numbers of rabbits are required to confirm these associations.

## Authors’ Contributions

KE and SA carried out the experiment design, participated in practical work, and wrote the manuscript. AE carried out the statistical analysis. KE had the primary responsibility for the content of the manuscript. All authors read and approved this manuscript.

## References

[1] Kapadiya D.B, Prajapati D.B, Jain A.K, Mehta B.M, Darji V.B, Aparnathi K.D (2016). Comparison of Surti goat milk with cow and buffalo milk for gross composition, nitrogen distribution, and selected minerals content. Vet World.

[2] Tyagi K.K, Brahmkshtri B.P, Ramani U.V, Kharadi V.B, Pandaya G.M, Janmeda M, Ankuya K.J, Patel M.D, Sorathiya L.M (2016). Test day variability in yield and composition of Surti and Mehsani buffaloes milk at day 15 and 60 postpartum. Vet. World.

[3] Estany J, Baselga M, Blasco A, Camacho J (1989). Mixed model methodology for the estimation of genetic response to selection in litter size of rabbits. Livest. Prod. Sci.

[4] El-Raffa A.M (2007). Formation of a rabbit synthetic line and primary analysis of its productive and reproductive performance. Egypt J Poult. Sci.

[5] Caroli A.M, Chessa S, Erhardt G.J (2009). Invited review: Milk protein polymorphisms in cattle: Effect on animal breeding and human nutrition. J. Dairy Sci.

[6] El-Sabrout K, Aggag S.A (2015). Use of inter simple sequence repeats and protein markers in assessing genetic diversity and relationships among four rabbit genotypes. World Rabbit Sci.

[7] AOAC (1990). Official Methods of Analysis.

[8] SPSS Statistical Package for the Social Sciences (2011). SPSS User's Guide: Statistics, Version 20.0 for Windows.

[9] DiMeo C, Gazaneo M.P, Racca1 C, Bovera F, Piccolo G, Nizza A (2004). Effect of birth weight and litter size on productive performance of rabbits. Asian Aust. J. Anim. Sci.

[10] Mahmoud E.A (2013). A study on some factors affecting milk yield in New Zealand white rabbits under Egyptian conditions. Benha Vet. Med. J.

[11] Kacsala L, Matics Z.S, Kasza R, Gerencsér Z.S, Szendrő Z.S (2015). Milk supply of rabbit kits. Poljo Privreda.

[12] Volek Z, Marounek M, Volková L, Kudrnová E (2014). Effect of diets containing whole white lupin seeds on rabbit doe milk yield and milk fatty acid composition as well as the growth and health of their litters. J. Anim. Sci.

[13] Abou Khadiga G, Youssef Y.M.K, Baselga M (2012). Characterization of reproductive performance of the APRI line of rabbits 10th.

[14] Chrenek P, Chrastinova L, Kirchnerova K, Makarevich A.V, Foltys V (2007). The yield and composition of milk from transgenic rabbits. Asian Aust. J. Anim. Sci.

[15] Maertens L, Vanacker J, De Coninck J (2006). Milk yield and milk composition of 2 commercial hybrids and a selected strain fed a high-energy lactation diet. 18^th^ Hungarian Conference on Rabbit Production, Kaposvar.

[16] Unsal D (2015). β-lactoglobulin genetic variants in brown-Swiss dairy cattle and their association with milk yield and quality traits. J. Anim. Plant Sci.

[17] Unsal D (2015). The influence of kappa casein protein polymorphism on milk production traits and other productive performance traits of brown-Swiss cattle. Pak. J. Zool.

[18] Ageitos J.M, Vallejo J.A, Poza M, Villa T.G (2006). Fluorescein thiocarbamoyl-kappa-casein assay for the specific testing of milk-clotting proteases. J. Dairy Sci.

